# Genomic Characterization of 16S rRNA Methyltransferase-Producing *Enterobacterales* Reveals the Emergence of *Klebsiella pneumoniae* ST6260 Harboring *rmtF*, *rmtB*, *bla*_NDM-5_, *bla*_OXA-232_ and *bla*_SFO-1_ Genes in a Cancer Hospital in Bulgaria

**DOI:** 10.3390/antibiotics13100950

**Published:** 2024-10-10

**Authors:** Stefana Sabtcheva, Ivan Stoikov, Sylvia Georgieva, Deyan Donchev, Yordan Hodzhev, Elina Dobreva, Iva Christova, Ivan N. Ivanov

**Affiliations:** 1 Laboratory for Clinical Microbiology, National Oncology Center, 1797 Sofia, Bulgaria; istoykov@sbaloncology.bg (I.S.); sigeorgieva@sbaloncology.bg (S.G.); 2 Department of Microbiology, National Center of Infectious and Parasitic Diseases, 1504 Sofia, Bulgaria; deyandonchev@ncipd.org (D.D.); y.hodzhev@ncipd.org (Y.H.); elina_dobreva@ncipd.org (E.D.); iva_christova@ncipd.org (I.C.); iivanov@ncipd.org (I.N.I.)

**Keywords:** aminoglycoside resistance, 16S rRNA methyltransferase, ArmA, RmtB, RmtF, *Enterobacterales*, *Klebsiella pneumoniae* ST6260

## Abstract

**Background:** Acquired 16S rRNA methyltransferases (16S-RMTases) confer high-level resistance to aminoglycosides and are often associated with β-lactam and quinolone resistance determinants. **Methods:** Using PCR, whole-genome sequencing and conjugation experiments, we conducted a retrospective genomic surveillance study of 16S-RMTase-producing *Enterobacterales*, collected between 2006 and 2023, to explore transmission dynamics of methyltransferase and associated antibiotic resistance genes. **Results:** Among the 10,731 consecutive isolates, 150 (1.4%) from 13 species carried *armA* (92.7%), *rmtB* (4.7%), and *rmtF* + *rmtB* (2.7%) methyltransferase genes. The coexistence of extended-spectrum β-lactamase (*bla*_CTX-M-3/15_, *bla*_SHV-12_, *bla*_SFO-1_), carbapenemase (*bla*_NDM-1/5_, *bla*_VIM-1/4/86_, *bla*_OXA-48_), acquired AmpC (*bla*_CMY-2/4/99_, *bla*_DHA-1_, *bla*_AAC-1_), and plasmid-mediated quinolone resistance (*qnrB*, *qnrS*, *aac(6′)-Ib-cr*) genes within these isolates was also detected. Methyltransferase genes were carried by different plasmids (IncL/M, IncA/C, IncR, IncFIB, and IncFII), suggesting diverse origins and sources of acquisition. *armA* was co-transferred with *bla*_CTX-M-3/15_, *bla*_NDM-1_, *bla*_VIM-4/86_, *bla*_OXA-48_, *bla*_CMY-4_, *aac(6′)-Ib-cr*, *qnrB*, and *qnrS*, while *rmtF1* was co-transferred with *bla*_SFO-1_, highlighting the multidrug-resistant nature of these plasmids. Long-read sequencing of ST6260 *K. pneumoniae* isolates revealed a novel resistance association, with *rmtB1* and *bla*_NDM-5_ on the chromosome, *bla*_OXA-232_ on a conjugative ColKP3 plasmid, and *rmtF1* with *bla*_SFO-1_ on self-transmissible IncFIB and IncFII plasmids. **Conclusions:** The genetic plasticity of plasmids carrying methyltransferase genes suggests their potential to acquire additional resistance genes, turning 16S-RMTase-producing *Enterobacterales* into a persistent public health threat.

## 1. Introduction

Aminoglycosides play an important role in the treatment of complicated bacterial infections, often used in synergistic combination with cell-wall-active antibiotics such as β-lactams. They act by specifically binding to the aminoacyl site (A-site) of 16S rRNA in prokaryotic 30S ribosomal subunits, thereby inhibiting protein synthesis with subsequent bacterial death [1]. Their widespread use has contributed to the emergence of aminoglycoside resistance involving target alteration, enzymatic drug modification, decreased permeability, and increased efflux [1,2]. Enzymatic drug inactivation through modification by aminoglycoside acetyltransferases, adenylyltransferases and phosphotransferases is the most common mechanism of aminoglycoside resistance in Gram-negative bacteria. Aminoglycoside-modifying enzymes have a well-defined substrate profile, including either amikacin or gentamicin, but never both, which favors a therapeutic approach. Unlike target modification, only the coexistence of various aminoglycoside-modifying enzymes in association with active efflux can result in high-level, broad-spectrum resistance.

Alteration of ribosomal targets by post-transcriptional methylation is an emerging mechanism, formerly confined to aminoglycoside producers as a mechanism of self-protection to avoid suicide [3]. The 16S rRNA methyltransferases (16S-RMTases) modify specific rRNA nucleotide residues within the A-site that blocks aminoglycosides from binding to their target and results in high-level resistance with a minimum inhibitory concentration (MIC) > 256 mg/L. These enzymes are divided into two classes depending on specific nucleotide residues that they modify. The methylation of the N-7 position of nucleotide G1405 by various 16S-RMTases confers resistance to 4,6-disubstituted 2-deoxystreptamines (kanamycin, amikacin, isepamicin, gentamicin, netilmicin, tobramycin, arbekacin, sisomicin, plazomicin), but not to 4,5-disubstituted (neomycin, ribostamycin, paromomycin) and 4-monosubstituted (apramycin) deoxystreptamines. In contrast, the methylation of the N-1 position of nucleotide A1408 confers resistance to deoxystreptamines of all groups. The methyltransferases of both classes are not active against atypical aminoglycosides (streptomycin, spectinomycin) that lack a deoxystreptamine ring [1,2,3]. The first cases of acquired plasmid-mediated 16S-RMTase in human pathogens were RmtA (ribosomal methyltransferases) in *Pseudomonas aeruginosa* from Japan [4], and ArmA (aminoglycoside resistance methyltransferase) in *Klebsiella pneumoniae* from France [5]. Subsequently, seven additional plasmid-borne methyltransferases, encoded by the genes *rmtB* [6], *rmtC* [7], *rmtD* [8], *rmtE* [9], *rmtF* [10], *rmtG* [11], and *rmtH* [12], have emerged in clinical isolates. They showed high-level resistance to clinically relevant aminoglycosides (i.e., amikacin, gentamicin, tobramycin, and plazomicin), but susceptibility to neomycin and apramycin. These enzymes were confirmed to function as N7-G1405 16S-RMTases [2,3]. In 2007, the first acquired N1-A1408 16S-RMTase was discovered. This enzyme, named NpmA, was identified in an *Escherichia coli* clinical strain in Japan. NpmA was plasmid-borne and confers resistance to neomycin and apramycin in addition to amikacin, gentamicin, tobramycin, and plazomicin [2,13].

Since their acquisition from clinical isolates of Gram-negative bacteria, 16S-RMTase genes have spread among *Enterobacterales*, *P. aeruginosa*, and *Acinetobacter baumannii* in both clinical and veterinary settings all over the world [14,15]. There are several features of 16S-RMTases that are involved in their dissemination and are of clinical concern. Most of the structural genes have been found in association with mobile genetic elements, such as broad- and narrow-host-range plasmids (e.g., IncL/M, IncA/C and IncF) [14,15] and transposons (e.g., Tn*1548* with *armA*) [16], suggesting that both conjugation and transposition are likely responsible for the acquisition and dissemination of these genes worldwide. Furthermore, 16S-RMTase genes are frequently associated with other antibiotic resistance genes of clinical importance, such as carbapenemase, extended-spectrum β-lactamase (ESBL) and plasmid-mediated quinolone resistance (PMQR) determinants. This could compromise the main classes of antimicrobials used to treat multi-resistant Gram-negative infections [3].

16S-RMTase-producing clinical isolates from Bulgaria were first reported in 2005 [16]. The reported isolates (two *Citrobacter freundii*, two *K. pneumoniae*, and one isolate each of *E. coli*, *Salmonella enterica*, and *Shigella flexneri*), collected in 2003, carried *armA* as part of the Tn*1548* transposon along with *bla*_CTX-M-3_ on IncL/M plasmids. Following this report, systematic surveillance for 16S-RMTase producers was introduced at the cancer hospital in Sofia. The results from the 2004–2005 study period showed that among 1310 *Enterobacterales*, 242 *P. aeruginosa* and 97 *A. baumannii,* the *armA* gene was identified in 20 (1.5%) *Enterobacterales* (7 *K. pneumoniae*, 3 *E. coli*, 3 *Serratia marcescens*, 3 *C. freundii*, 3 *Enterobacter cloacae*, and 1 *Klebsiella oxytoca*). ArmA-mediated aminoglycoside resistance was transferable by conjugation and carried by closely related IncL/M plasmids, which also carried *aadA2*, *dfrA12*, *sul1*, *bla*_TEM-1_, and *bla*_CTX-M-3_ genes encoding resistance to streptomycin, trimethoprim, sulfonamides, and ß-lactams, respectively. Most of the isolates were genetically different, but shared similar restriction patterns of the *armA*-encoding plasmids [17]. Based on these findings, the systematic surveillance continued.

In this study, we conducted a retrospective genomic surveillance study of 16S-RMTase-producing *Enterobacterales* at a Bulgarian hospital from 2006 to 2023, aiming to improve our understanding of the transmission dynamics of methyltransferase genes and their association with other antibiotic resistance determinants.

## 2. Results

### 2.1. Identification of 16S rRNA Methyltransferase-Producing Bacterial Isolates

**Figure 1:**
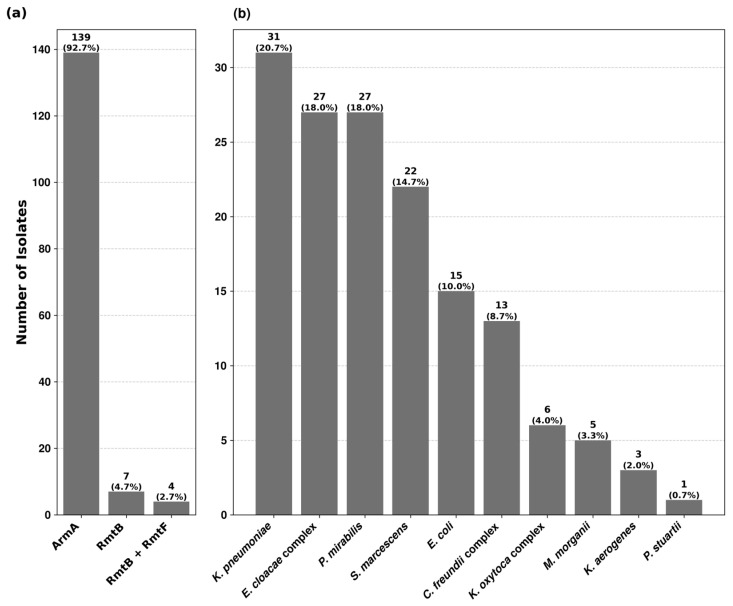
Prevalence of 16S rRNA methyltransferase genes and their distribution among 16S-RMTase-producing isolates. (**a**) The first chart shows the prevalence rate of ArmA, RmtB and RmtF methyltransferases among the 150 bacterial isolates. (**b**) The second chart depicts the distribution of 16S-RMTase-producing isolates across the studied bacterial species.

Among the 10,731 isolates, tested for high-level resistance to aminoglycosides, 150 (1.4%) showed concomitant resistance to gentamicin and amikacin with no zone of inhibition around the discs and MICs > 256 mg/L. No isolate was found with high-level resistance to apramycin. PCR analysis revealed the presence of 16S rRNA methyltransferase genes in all 150 isolates (Table S1). 16S-RMTase producers were distributed among thirteen *Enterobacterales* species. The prevalence rates within each species varied from 0.3% to 16.7%, being the highest in *Providencia stuartii* (Table S2). Three types of methyltransferase genes were identified, of which *armA* was the most predominant, followed by *rmtB* and *rmtF*. Their distribution among different bacterial isolates is depicted in Figure 1.

The most common isolation site was urine (83%, 124/150), followed by wound exudates (13%, 20/150), respiratory samples (3%, 4/150), blood (1/150) and abdominal fluid (1/150). Further information for each bacterial species can be found in Table S1.

### 2.2. Identification of β-Lactamase and Plasmid-Mediated Quinolone Resistance Genes

**Figure 2:**
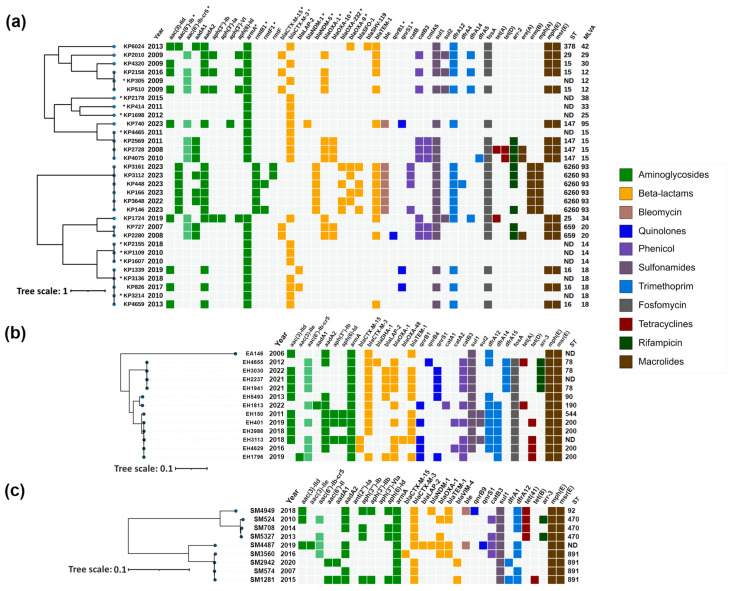
Heatmap representation of the acquired antimicrobial resistance genes of (**a**) *Klebsiella pneumoniae*; (**b**) *Enterobacter cloacae* complex; (**c**) *Serratia marcescens*. Phylogenetic trees were constructed based on SNP analysis for (**b**,**c**) with PhaME v1.0.4, while for (**a**), MLVA types were used for phylogenetic tree construction according to Grissa et al. [18] (see Section 4.7). For (**a**) strains marked with an asterisk (*), resistance data were obtained from PCR and for the remaining strains from WGS. Drug classes are colored consistently, as shown in the legend (right). The only exception from the legend is *aac(6′)-Ib-cr5*, colored in pale green to indicate dual resistance. Both dendrograms and heatmaps were annotated with iTOL v6.8.1 (https://itol.embl.de/about.cgi, accessed on 20 August 2024). Sequence type (ST) was derived for the species with available MLST schemes. ND, not determined either due to missing alleles or imperfect match. KP, *Klebsiella pneumoniae*; EA, *Enterobacter asburiae*; EH, *Enterobacter hormaechei*; SM, *Serratia marcescens*.

**Figure 3:**
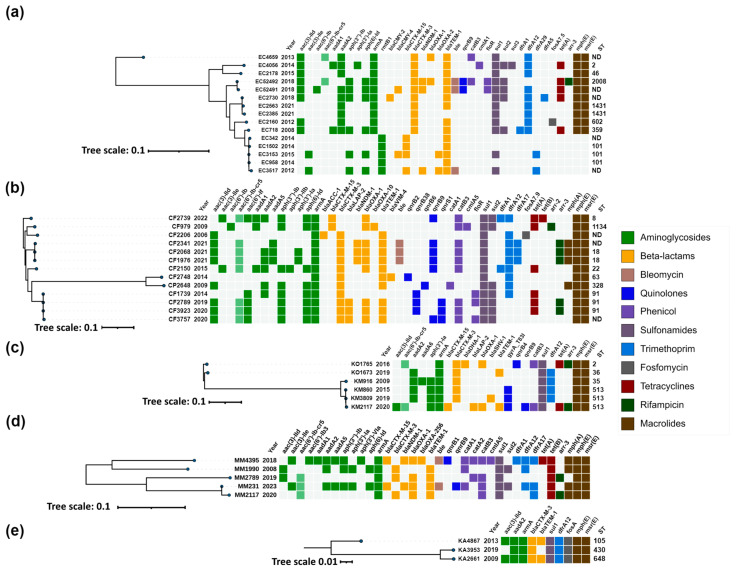
Heatmap representation of the acquired antimicrobial resistance genes of (**a**) *Escherichia coli*; (**b**) *Citrobacter freundii* complex; (**c**) *Klebsiella oxytoca* complex; (**d**) *Morganella morganii*; (**e**) *Klebsiella aerogenes*. Phylogenetic trees were constructed based on SNP analysis for (**a**–**e**) with PhaME v1.0.4 (see Section 4.7). Drug classes are colored consistently, as shown in the legend (right). The only exception from the legend is *aac(6′)-Ib-cr5*, colored in pale green to indicate dual resistance. Both dendrograms and heatmaps were annotated with iTOL v6.8.1 (https://itol.embl.de/about.cgi, accessed on 20 August 2024). Sequence type (ST) was derived for the species with available MLST schemes. ND, not determined either due to missing alleles or imperfect match. EC, *Escherichia coli*; CF, *Citrobacter freundii*; CP, *Citrobacter portucalensis*; KO, *Klebsiella oxytoca*; KM, *Klebsiella michiganensis*; MM, *Morganella morganii*; KA, *Klebsiella aerogenes*.

ESBL genes were identified in 97% (145/150) of the 16S-RMTase-producing isolates, with *bla*_CTX-M-3_ (67%, 97/145) being the most common, followed by *bla*_SHV-12_ (19%, 27/145), *bla*_CTX-M-15_ (12%, 17/145) and *bla*_SFO-1_ (3%, 4/145) (Table S1, Figure 2 and Figure 3).

Carbapenemase genes were identified in 33% (49/150) of the 16S-RMTase-producers. WGS analysis identified *bla*_VIM-1/4/86_ allelic variants in 63% (31/49), *bla*_NDM-1/5_ in 35% (17/49) and *bla*_OXA-48_ in a single *E. hormaechei* strain.

Acquired AmpC enzymes were identified in 23% (34/150) of the 16S-RMTase-producing isolates, including *bla*_CMY-99_ (*n* = 27), *bla*_CMY-4_ (*n* = 3) and *bla*_DHA-1_ (*n* = 2). *bla*_CMY-2_ and *bla*_AAC-1_ genes were also detected in one isolate each.

The plasmid-mediated fluoroquinolone resistance gene *aac(6′)-Ib-cr* was identified in 36% (54/150) of the 16S-RMTase-producing isolates of almost all species, except *P. mirabilis* and *K. aerogenes*, while *qnrB* variants and *qnrS1* were found in 17% and 9%, respectively.

### 2.3. Exploring Genomic Diversity of 16S rRNA Methyltransferase-Producing Isolates

Genomic characteristics, correlation between acquired antimicrobial resistance genes, year of isolation, MLST profiles (where applicable), and phylogenetic relatedness of isolates within each species are illustrated in Figure 2 and Figure 3.

#### 2.3.1. *Klebsiella pneumoniae*

*K. pneumoniae* isolates were distributed across six main clusters corresponding to sequence types (STs) ST15, ST147, ST6260, ST659 and ST16, in addition to MLVA type 14, for which STs remained undetermined (Figure 2a).

The ST15 cluster comprised four isolates, three from 2009 and one from 2016. All isolates carried *armA*, with two (KP4320 and KP305) harboring *bla*_CTX-M-3_, while the other two (KP2158 and KP510) contained *bla*_CTX-M-15_.

The ST147 cluster included five isolates, collected between 2008 and 2023, all exhibiting *armA* and *bla*_CTX-M-3_. Notably, one isolate, KP740, also harbored the carbapenemase gene *bla*_NDM-1_, and the quinolone resistance genes *qnrS1* and *aac(6′)-Ib-cr5*.

*K. pneumoniae* ST6260 exhibited a concerning array of resistance genes. Long-read sequencing analysis revealed that *rmtB1* and *bla*_NDM-5_, which were present in all isolates, were chromosomally encoded. Additionally, four isolates carried *rmtF* genes; two were identified as *rmtF1*, while the remaining two variants remained undetermined. Manual inspection of the sequences suggested that these variants are most likely also *rmtF1*. The *rmtF* genes were hosted on IncFIB(K)-type plasmids, with some of these being multireplicon plasmids that also contained IncFIB(pQil) and IncFII(pKP91) replicons. These plasmids in some isolates also harbored the *bla*_SFO-1_ and *bla*_OXA-9_ β-lactamase genes. Furthermore, the *bla*_OXA-232_ carbapenemase gene was detected in four of these isolates, situated on a small ~6141 bp plasmid of ColKP3 type. *rmtF*, *bla*_SFO-1_, *bla*_OXA-9_ and *bla*_OXA-232_ were successfully transferred by conjugation, and were confirmed by PCR (Table 1).

The ST659 cluster included two isolates from 2007 and 2008, both carrying *armA*, *bla*_CTX-M-15_, *bla*_OXA-1_, *bla*_OXA-10_ and *aac(6′)-Ib-cr5*, with one isolate (KP2280) additionally harboring *qnrB1*.

The MLVA type 14 cluster consisted of three isolates collected in 2008 (two isolates) and 2018 (one isolate), all possessing *armA* and *bla*_CTX-M-3_.

The ST16 cluster, with isolates collected between 2010 and 2019, consistently harbored *armA*, with most isolates also containing *bla*_CTX-M-3_, except for KP1339. Two isolates, KP1339 and KP826, also possessed *qnrS1*.

The remaining isolates outside these clusters, though unrelated, included KP6024, KP2178, KP414, KP1698, KP1724, and KP2010 and all carried *armA*, with most harboring *bla*_CTX-M-3_ except for KP2010, which carried *bla*_CTX-M-15_. KP6024 also carried *bla*_OXA-9_.

Transfer of high-level aminoglycoside resistance was achieved from 24/25 *K. pneumoniae* clinical strains. The *armA*-positive transconjugants always carried *bla*_CTX-M-3_ or *bla*_CTX-M-15_ and to a lesser extent, *bla*_OXA-1_, *bla*_OXA-10_, *aac(6′)-Ib-cr*, and *qnrB1* (Table 2). PCR-based replicon typing (PBRP) showed that *armA* was mainly carried by IncL/M plasmids. However, in a transconjugant obtained from KP2158, co-carriage of *armA* and *bla*_CTX-M-15_ on an IncR plasmid was detected, while three plasmids harboring *armA* and *bla*_CTX-M-3_, and three others with *armA* and *bla*_CTX-M-15_ could not be typed with the PBRT method.

#### 2.3.2. *Enterobacter cloacae* Complex

Most of the isolates were identified as *Enterobacter hormaechei*, except EA146, which was characterized as *Enterobacter asburiae.* The presence of *armA* and *bla*_CTX-M_ genes was detected across all isolates (Table S1). PCR and sequencing identified CTX-M-3 ESBL in 25/27 isolates. *bla*_OXA-1-like_, and quinolone resistance genes *aac(6′)-Ib-cr* and *qnr* were detected in 15/27, 16/27 and 13/27 ArmA-producers, respectively. The only carbapenemase, OXA-48-like, was found in the *E. hormaechei* strain EH3113. BOX-PCR identified eight genotypes (Table S1). Based on PCR results and molecular typing, thirteen isolates were selected for WGS. These included EH3113 (*bla*_OXA-48-like_-positive), EH4629 (*bla*_CTX-M-3_-negative), ten *E. hormaechei* isolates with different genetic configurations and genotypes, and the *E. asburiae* strain EA146.

*E. asburiae* 146, collected in 2006, carried *armA* along with *bla*_CTX-M-3_, *aadA2*, *dfrA12* and *sul1* genes (Figure 2b), similar to the genetic context of *armA* on the IncL/M pCTX-M3 [19]. Among the *E. hormaechei* isolates, subjected to WGS analysis, four isolates from the period 2012–2022 formed a cluster corresponding to ST78. The earliest isolate from this cluster (EH4655) carried *qnrB4*, while subsequent isolates harbored *qnrS1*. In addition, EH4655 had an acquired *bla*_DHA-1_ AmpC gene. All isolates in this cluster also carried *bla*_OXA-1_ and *aac(6′)-Ib-cr5* along with *armA* and *bla*_CTXM-3_ genes.

Another cluster, covering the years 2016 to 2019 and corresponding to ST200, consisted of five isolates. Within this group, three isolates were identified with *bla*_CTX-M-3_, and two with *bla*_CTX-M-15_. The presence of the carbapenemase gene, *bla*_OXA-48_, was also noted in EH3113. Four isolates from this cluster carried the *qnrB1* gene, with one additionally harboring *qnrS1*.

The remaining three isolates were associated with different sequence types (ST90, ST190, and ST544), and exhibited a similar acquired antimicrobial resistance gene profile with the presence of *armA* and *bla*_CTX-M-3_. Among these, isolates EH5493 and EH1813 were noted for carrying *qnrS1* and *qnrB1*, respectively.

*armA*-positive transconjugants were obtained from 26/27 clinical isolates. In EH3113, co-transfer of *armA* with *bla*_OXA-48_, *bla*_OXA-1_, *aadA2*, *qnrB1* and *aac(6′)-Ib-cr* on IncL/M plasmids was observed (Table 1). In the remaining transconjugants, *armA* was consistently located on CTX-M-3 IncL/M plasmids, which were often associated with additional resistance genes such as *bla*_OXA-1_ and *aac(6′)-Ib-cr* (Table 2).

#### 2.3.3. *Serratia marcescens*

PCR analysis detected the presence of *armA* and *bla*_CTX-M_ genes in all isolates (Table S1). In addition, *bla*_VIM_ or *bla*_NDM_ were detected in 2 isolates each, *bla*_OXA-1-like_ and *aac(6′)-Ib-cr* were identified in 10/22, and *qnrB* or *qnrS* in single strains. CTX-M-3 was confirmed in 21/22 isolates. BOX-PCR typing distinguished eight genotypes (Table S1). Based on the PCR and genotyping data, nine isolates were selected for WGS.

*S. marcescens* isolates, subjected to WGS analysis, were distributed across three sequence types: ST92, ST470, and ST891, with each isolate carrying *armA* and *bla*_CTX-M_ genes (Figure 2c). The ST92 group included a single isolate from 2018 that exhibited a diverse array of resistance genes, including *armA*, *bla*_CTX-M-3_, *bla*_NDM-1_, and *qnrB9*. Isolates in the ST470 group, collected between 2010 and 2014, uniformly displayed the presence of *armA* and *bla*_CTX-M-3_. In the ST891 cluster, all isolates harbored *armA*, with most also carrying *bla*_CTX-M-3_; this cluster included SM3560 with CTX-M-15 and two other isolates with VIM-4 carbapenemase. Additionally, a single isolate with an undetermined ST, collected in 2019, was found to harbor *armA*, *bla*_CTX-M-3_, *bla*_NDM-1_, and *qnrS1*.

*armA* was transferred by conjugation from all donor strains and was carried by IncL/M plasmids along with *bla*_CTX-M-3_ or *bla*_CTX-M-15_, and in a single case, with *bla*_OXA-1_ and *aac(6′)-Ib-cr* (Table 2). In a transconjugant, obtained from SM5327, co-carriage of *armA* and *bla*_CTX-M-3_ on IncA/C plasmids was observed. Carbapenemase genes were not co-transferred with *armA*, except for SM4949, whose transconjugant harbored *armA* with *bla*_CTX-M-3_, *bla*_NDM-1_, *aadA2* and *qnrB9* on an IncL/M plasmid (Table 1).

#### 2.3.4. *Escherichia coli*

Two main resistance genes profiles were determined for *E.coli* isolates, including one with *armA* and *bla*_CTX-M-3_, and one with *rmtB* and *bla*_CTX-M-15_ (Figure 3a). The first profile was presented by two clusters corresponding to ST2008 and ST1431, as well as three unrelated isolates collected between 2013 and 2015, and two additional isolates from 2012 and 2008 with ST602 and ST359, respectively. The isolates from the ST2008 cluster were collected in 2018 and were associated with the presence of *bla*_NDM-1_ and *qnrB9*. Additionally, one of the isolates (EC52491) also carried *bla*_CMY-4_, while the other isolate (EC52492) carried *bla*_OXA-1_ and *aac(6′)-Ib-cr5*. The isolates from the ST1431 cluster harbored the classical configuration of *armA* and *bla*_CTX-M-3_, and only one of these isolates (EC2730) from 2018 also contained *bla*_CMY-2_. The unrelated isolates collected between 2008 and 2015 from the first profile shared the same configuration of *armA* and *bla*_CTX-M-3_, with one of these isolates (EC4659) from 2013 additionally harboring *bla*_OXA-1_ and *aac(6′)-Ib-cr5*.

The second profile was presented with one cluster corresponding to ST101 and comprised isolates collected between 2012 and 2015. All isolates harbored *rmtB1*, and all except one (EC958) had *bla*_CTX-M-15_ genes. In addition, one isolate (EC3517) harbored the carbapenemase gene *bla*_NDM-1_, while another isolate from 2015 (EC3153) carried the *bla*_CMY-4_ AmpC gene.

All isolates harboring ArmA and CTX-M-3 yielded transconjugants, which were positive for *armA* and *bla*_CTX-M-3_ on IncL/M plasmids (Table 2). Co-transfer of *armA* with *bla*_CTX-M-3_, *bla*_NDM-1_, *bla*_CMY-4_, and *qnrB9* on IncL/M plasmids was observed in EC52491 (Table 1). In contrast, two transconjugants were obtained from EC52492, including one harboring *armA*, *bla*_CTX-M-3_, *bla*_OXA-1_ and *aac(6′)-Ib-cr* on IncL/M plasmids, and another carrying *bla*_NDM-1_ and *qnrB9* on IncA/C plasmid.

Conjugal transfer of *rmtB1* from *E. coli* donor strains could not be achieved despite repeated attempts. However, WGS analysis of donors’ plasmid replicons and in silico plasmid analysis showed that in EC3517, EC342 and EC3153, the *rmtB1* gene was located on IncFII, IncA/C and IncFII plasmids, respectively. In EC958 and EC1502, the plasmid location of *rmtB1* genes was suspected, but could not be proven by the only short-read sequencing data available.

#### 2.3.5. *Citrobacter freundii* Complex

The isolates of the *C. freundii* complex, including *C. freundii* strains and single isolate of *Citrobacter portucalensis*, were characterized by the consistent presence of *armA* and *bla*_CTX-M_ genes (Figure 3b). They exhibited diverse sequence types, with two clusters being clearly distinguished: one corresponding to ST18 and the other to ST91. Outside of these clusters, the isolates displayed unique STs, indicating a lack of relatedness.

The ST18 cluster comprised isolates collected in 2021, all of which were positive for *armA*, *bla*_CTX-M-3_, and also harbored the carbapenemase gene *bla*_NDM-1_ along with *qnrB9* and *aac(6′)-Ib-cr5* quinolone resistance genes. The ST91 cluster also exhibited the ArmA/CTX-M-3 configuration, accompanied by various *qnr* genes. One isolate from this cluster, CF1739 (2014), carried only *qnrB38*. Other isolates within this cluster included CF2789 and CF3923, which harbored *qnrB38* in combination with *qnrS1*, and CF3757, which carried both *qnrB9* and *qnrS1* in addition to *aac(6′)-Ib-cr5*.

Two isolates, CF2739 (2022) and CF979 (2009), were characterized by the presence of *bla*_CTX-M-15_ instead of *bla*_CTX-M-3_, with CF2739 also harboring *qnrB9* and *aac(6′)-Ib-cr5*. The earliest isolate in this panel, CF2206 (2006), possessed *armA*, *bla*_CTX-M-3_, and *bla*_ACC-1_. Other notable isolates included CF2150, CF2748, and CP2648, all of which had the ArmA/CTX-M-3 profile. CF2150 additionally harbored *qnrB9* and *aac(6′)-Ib-cr5*, while CP2648 had *qnrB6*. CF2748 was distinguished by the production of VIM-4 metallo-β-lactamase and *qnrB2*.

*armA* was successfully transferred from all donors, yielding transconjugants with varied genetic contents, but always positive for *bla*_CTX-M-3_ or *bla*_CTX-M-15_, and *bla*_NDM-1_ or *bla*_VIM-4_ when present in the donors (Table 1 and Table 2). PBRT confirmed that *armA* was located on IncL/M plasmids in all transconjugants, except one obtained from CF2739, in which co-carriage of *armA* and *bla*_CTX-M-15_ on the IncFIB plasmid was observed (Table 2).

#### 2.3.6. *Klebsiella oxytoca* Complex

The isolates of the *K. oxytoca* complex were predominantly identified as *Klebsiella michiganensis*, with the exception of two isolates that were classified as *K. oxytoca*. All isolates within this complex harbored *armA* and *bla*_CTX-M_ genes (Figure 3c). The two *K. oxytoca* isolates were characterized by the ArmA/CTX-M-3 configuration and one of them, KO1765, also carried *bla*_DHA-1_, *qnrB4* and *aac(6′)-Ib-cr5* genes.

The *K. michiganensis* group included one isolate from 2009, identified as ST35, and three isolates collected between 2015 and 2020, which shared the sequence type ST513 and formed a distinct cluster. Most *K. michiganensis* isolates exhibited the ArmA/CTX-M-3 profile. An exception was the isolate KM2117, which harbored *bla*_CTX-M-15_ instead of *bla*_CTX-M-3_, and also carried *qnrB9* and *aac(6′)-Ib-cr5* quinolone resistance genes.

Thansconjugants carrying *armA* were obtained from all clinical isolates. The *armA* gene was consistently located on IncL/M plasmids, which also carried *bla*_CTX-M-3_ or *bla*_CTX-M-15_, and variably *aadA2*, *bla*_OXA-1_ and *aac(6′)-Ib-cr* genes (Table 2).

#### 2.3.7. *Morganella morganii*

*M. morganii* isolates, though unrelated, consistently harbored *armA* and *bla*_CTX-M_ genes (Figure 3d). The majority carried *bla*_CTX-M-3_, with the exception of two isolates, MM4395 and MM231, which exhibited *bla*_CTX-M-15_. Additionally, MM4395 and MM231 were notable for also having the NDM-1 carbapenemase and *qnrB* genes, specifically *qnrB1* in MM4395 and *qnrB9* in MM231, along with *aac(6′)-Ib-cr5.*

*ArmA* was transferred in all but one isolate. In transconjugants from MM1990, MM2789 and MM2117, *armA* was carried by CTX-M-3 IncL/M plasmids with various associated resistance genes (Table 2). In MM4395, a co-transfer of *armA* with *bla*_NDM-1_, *bla*_OXA-1_, *qnrB1* and *aadA2* on IncT plasmids was observed (Table 1). In contrast, no plasmid replicons were detected in MM231, and it failed to transfer any resistance determinants by conjugation, suggesting the chromosomal location of *armA* and associated resistance genes.

#### 2.3.8. *Klebsiella aerogenes*

*K. aerogenes* isolates were distributed across three distinct sequence types—ST105, ST430, and ST648—all of which possessed the *armA*, *bla*_CTX-M-3_, *aadA2*, *dfrA12*, and *sul1* genes typical for the genetic context of *armA* on the IncL/M pCTX-M3 plasmid [19], and without other resistance genes (Figure 3e).

Conjugal transfer of *ArmA* was achieved from all donor strains. *armA* was always carried by IncL/M plasmids along with *bla*_CTX-M-3_ and *aadA2* genes (Table 2).

#### 2.3.9. *Proteus mirabilis*

Molecular typing was performed sequentially on 27 *P. mirabilis* isolates collected between 2007 and 2021 (Table S1). First, the isolates were identified as belonging to the Pm-1 “Dienes strain” as judged by the Dienes test. PFGE typing of selected isolates from each year showed the presence of identical patterns, suggesting that all isolates belonged to a single clone, which had already been found for ten of the isolates described previously. [20]. PCR assays detected the presence of *armA* and identical co-harboring genes in all isolates (Table S1). WGS analysis of the representative strain PM1502, collected in 2014, revealed several resistance genes including *armA*, *aac(6′)-Ib*, *aadA1*, *aadA2*, *aph(3″)-Ib*, *aph(6)-Id* (aminoglycoside resistance), *bla*_VIM-1_, *bla*_CMY-99_, *bla*_SHV-12_, *bla*_OXA-9_, *bla*_TEM-1_ (β-lactam resistance), *dfrA1*, *dfrA12* (trimethoprim resistance), and *sul1*, *sul2* (sulfonamide resistance).

Conjugal transfer of *armA* from clinical isolates of *P. mirabilis* could not be achieved and no plasmid replicons were detected in PM1502, suggesting chromosomal location of *armA* and associated resistance genes. However, this was not investigated further as PM1502 had only short-read sequencing data available.

#### 2.3.10. *Providentia stuartii*

The only ArmA-producing *P. stuartii* strain, PS3347, was identified in 2020 (Table S1). This strain was previously described in our study on carbapenem-resistant AmpC producers [21]. PS3347 harbored a diverse array of resistance genes located on an IncA/C conjugative plasmid, including *armA*, *bla*_VIM-86_, *bla*_CMY-4_, *bla*_OXA-1_, and *aac(6′)-lb-cr5* (Table 1).

### 2.4. Antimicrobial Susceptibility of 16S rRNA Methyltransferase-Producing Isolates

The results of susceptibility testing for each bacterial isolate, along with the resistance genes detected, are shown in Table S4. All isolates were concomitantly highly resistant to amikacin and gentamicin, consistent with the presence of 16S rRNA methyltransferase genes. In addition, almost all isolates were resistant to cephalosporins and most isolates to fluoroquinolones, due to ESBLs with or without carbapenemases and quinolone resistance determinants, respectively. All but two isolates (PS3347 and KP3112) were susceptible to cefiderocol, whereas resistance to ceftazidime–avibactam, imipenem–relebactam and meropenem–vaborbactam was associated with the presence of methalo-β-lactamase genes.

## 3. Discussion

This retrospective genomic surveillance study, conducted between 2006 and 2023 in a single hospital, is the first systematic evaluation of 16S rRNA methyltransferase-producing *Enterobacterales* in Bulgaria. The period prevalence was 1.4% (150/10,731), which is consistent with a previous study conducted at the same hospital that identified methyltransferase genes in 1.5% (20/1310) of enterobacterial isolates collected in 2004–2005 [17]. Our results, which show persistence of 16S-RMTase-mediated resistance despite infection control measures [22], suggest that the dissemination of methyltransferase genes was likely due to horizontal transmission rather than clonal spread. The prevalence comparison with analogous studies showed that Bulgaria has a lower prevalence rate than India [23] and Iran [24] where rates of 46.3% (57/123) and 13.0% (40/307) were reported in consecutive isolates, although the small number of isolates may not reflect the true prevalence rate. A similar prevalence rate of 1.1% (20/1770) was observed in Poland [25], whereas in the UK, 0.1% of the 56,172 *Enterobacterales* isolates collected in 2016 harbored methyltransferase genes [26].

We characterized three types of 16S rRNA methyltransferases genes. Among them, *armA* was the predominant type (92.7%), whereas *rmtB1* (4.7%) and *rmtF1* + *rmtB1* (2.7%) were only infrequently identified. *armA* was widely distributed in *Enterobacterales*, comprising thirteen species, while other methyltransferase genes were more species-specific, such as *rmtF*, which was found only in *K. pneumoniae*, and *rmtB* in *E. coli* and *K. pneumoniae*. During the study period, further spread of ArmA involving isolates of *P. mirabilis*, *M. morganii*, *P. stuartii* and *K. aerogenes* was observed. Furthermore, isolates with *rmtF* and *rmtB* genes were identified in our hospital, with *rmtF* being reported for the first time in Bulgaria. Our results confirm the researchers’ findings that ArmA and RmtB are the two most frequently reported 16S RMTases, and at the same time reveal the specificity of their distribution within the hospital [3,14].

16S rRNA methyltransferases were often reported to coexist with β-lactamase genes. A high percentage of 16S-RMTase-producing *Enterobacterales*, in our study, harbored ESBL genes (97%), with *bla*_CTX-M-3_ being the most common, which is in accordance with our previous studies [16,17]. In addition, during the study period, associations of *bla*_CTX-M-15_ with *armA* or *rmtB* in many species, and *bla*_SFO-1_ with *rmtF* and *rmtB* in *K. pneumoniae*, were identified using WGS analysis. Although associations of *bla*_CTX-M-15_ with *armA* and *rmtB* have been reported previously [27,28], as well as one case of *E. coli* possessing *bla*_SFO-1_ with *rmtB* [29], the coexistence of *bla*_SFO-1_ with *rmtF*, as far as we are aware, has not been reported to date. One-third of the 16S RMTase-producing *Enterobacterales* were also found to carry carbapenemase genes, with *bla*_VIM_ and *bla*_NDM_ being dominant. The carbapenemases NDM-5 and OXA-232 were found only in isolates of *K. pneumoniae* ST6260. VIM-1 was detected exclusively in isolates belonging to the *P. mirabilis* clone, while NDM-1 was widely distributed among different species. Half of these isolates belonged to the *Enterobacter* spp., *S. marcescens*, *C. freundii*, *Providencia* spp., and *M. morganii* (ESCPM) group and were previously described in our study on carbapenemase-producing enterobacteria with natural AmpC enzymes [21]. These results are in agreement with globally reported findings showing that 16S RMTase genes are commonly associated with carbapenemase genes [23,30,31]. Other antibiotic resistance genes frequently associated with 16S-RMTase genes in this study were the plasmid-mediated quinolone resistance determinants *aac(6′)-Ib-cr* (36%) and *qnr* (26%), whose co-existence with carbapenemase, ESBL, *armA* and *rmtB* has already been reported [27]. These associations lead to co-selection of antibiotic resistance genes by numerous antimicrobials and compromise the clinical use of the three main groups of bactericidal antibiotics for the treatment of life-threatening bacterial infections.

In this study, 16S rRNA methyltransferases were found to be associated with broad-host-range plasmids of diverse incompatibility groups, reflecting multiple sources of acquisition through horizontal gene transfer. In total, 116 isolates (77%) were found to carry the methyltransferase gene on a plasmid. Of these, one hundred and nine isolates of thirteen bacterial species harbored *armA* on at least five plasmid types, with IncL/M being the dominant plasmid type found. The majority of IncL/M plasmids carrying *armA* also carry *bla*_CTX-M-3_, similar to the IncL/M plasmid pCTX-M3 in *C. freundii* [19] and pIP1204 in *K. pneumoniae* [5], on which the *armA* gene was initially identified. While neither carbapenemase nor plasmid-mediated quinolone resistance genes were detected in our previous studies [16,17], in this study, various carbapenemase (*bla*_NDM-1_, *bla*_VIM-4_, *bla*_VIM-86_, *bla*_OXA-48_), PMQR (*aac(6′)-Ib-cr*, *qnrS*, *qnrB*), and other β-lactamase (*bla*_CMY-4_, *bla*_OXA-1_, *bla*_OXA-10_) genes were found on IncL/M plasmids co-transferred with *armA*. The *bla*_CTX-M-15_ gene instead of *bla*_CTX-M-3_ was also detected in *armA*-carrying IncL/M plasmids from some isolates. Furthermore, *armA* was found on IncA/C, IncR, IncFIB and IncT self-transmissible plasmids co-transferred with either *bla*_CTX-M-15_/*bla*_CTX-M-3_ or *bla*_VIM-86_/*bla*_NDM-1_ and other resistance genes.

*armA* was frequently co-located with *bla*_NDM-1_ or other carbapenemase genes (especially *bla*_VIM-86_, *bla*_VIM-4_ and *bla*_OXA-48_) on the same plasmids, with some of them additionally carrying genes for fluoroquinolone resistance, such as *aac(6′)-Ib-cr* and *qnrB*. The acquisition of these multidrug-resistant plasmids would have led to simultaneous resistance to most β-lactams, including carbapenems, as well as aminoglycosides and fluoroquinolones, the three major groups of antimicrobial agents active against Gram-negative bacteria.

A few high-risk clones were found to be associated with the ArmA gene, including *K. pneumoniae* ST147, *C. freundii* ST18 and a *P. mirabilis* clone endemic to Bulgaria, all of which have been reported to carry carbapenemase genes, such as *bla*_NDM-1_ in *K. pneumoniae* ST147 [32] and *C. freundii* ST18 [33], and *bla*_VIM-1_ in the *P. mirabilis* clone [34]. Besides these clones, an abundance of minor STs in ArmA-producing *Enterobacterales* was found in this study. On the basis of the clonal diversity observed by MLST and MLVA, it can be assumed that the persistence of methyltransferase genes in our hospital is driven by plasmid transmission rather than clonal spread.

*rmtB* was initially identified on a plasmid in association with *bla*_TEM-1_ in a clinical isolate of *S. marcescens* from Japan [6]. In this study, *rmtB1* was identified in five *E. coli* strains of the high-risk ST101 clone, which emerged as sporadic cases during a limited period (2012–2015). *rmtB* was found on non-conjugative IncFII and IncA/C plasmids in 3/5 isolates, which could explain the lack of further dissemination. The only *bla*_NDM-1_-positive strain, EC3517, appeared to be similar to the reported outbreak strain in another Bulgarian hospital in 2012 as both ST101 strains harbored *bla*_CTX-M-15_ in addition to *bla*_NDM-1_ and *rmtB*. However, while we identified *rmtB* on a non-conjugative IncFII plasmid, the transconjugants obtained from the outbreak strains were found to carry *rmtB* with *bla*_NDM-1_ on an untypable plasmid [35].

The most important finding of this study was the identification of *K. pneumoniae* ST6260 in our hospital, which was found to emerge in 2022 (Table S1). Long-read sequencing revealed a novel resistance association of RmtF1 and RmtB1 methyltransferases, NDM-5 and OXA-232 carbapenemases, and SFO-1 ESBL. The *rmtB1* and *bla*_NDM-5_ were chromosomally located in all isolates. The *rmtF1* was hosted on conjugative IncFIB(K) plasmids, with some of these being multireplicon plasmids that also contained IncFIB(pQil) and IncFII(pKP91) replicons. The *bla*_OXA-232_ carbapenemase gene was situated on a small ~6141 bp plasmid of ColKP3 type that could be transferred by conjugation. The *bla*_SFO-1_ ESBL gene was plasmid-born and co-transferred with *rmtF1*. In contrast to RmtB1 and NDM-5, plasmid-encoded RmtF1, OXA-232 and SFO-1 were variously present in some of the six investigated isolates. Based on the patient metadata (Table S1), which indicates that the first isolate of *K. pneumoniae* ST6260, KP3746, originated from an outpatient and the reported nosocomial spread of *K. pneumoniae* ST6260 in another Bulgarian hospital [36], it can be assumed that *K. pneumoniae* ST6260 is already spreading between hospitals in Bulgaria. Therefore, a national genome-wide survey is currently underway to determine the extent of the emerging clonal spread of *K. pneumoniae* ST6260 and to prevent its further expansion.

## 4. Materials and Methods

### 4.1. Bacterial Isolates, Identification and Susceptibility Testing

From January 2006 to October 2023, a total of 10,731 consecutive, nonduplicate enterobacterial isolates were collected from patients’ specimens in a 252-bed oncology hospital in Sofia, Bulgaria. Clinical specimens originated from infected or colonized patients admitted to hospital wards or attending outpatient departments. Bacterial isolates were initially identified by using GNI cards on the VITEK^®^2 system (bioMérieux, Marcy l’Étoile, France). The selected high-level aminoglycoside-resistant isolates were re-identified by MALDI-TOF Biotyper (Bruker Daltonics GmbH, Bremen, Germany) with MALDI Reference 2022 Library v.4.0.

Antimicrobial susceptibility testing was carried out by broth microdilution using the MicroScan NM-EN52 panel (Beckman Coulter, Inc., Brea, CA, USA) by following the manufacturer’s protocol. Susceptibility to cefiderocol, ceftazidime–avibactam, imipenem–relebactam, and meropenem–vaborbactam was determined by the disk diffusion method with MASTDISCS^®^ (Mast group, Merseyside, UK). Susceptibility testing results were interpreted in accordance with EUCAST clinical breakpoints v13.0. *E. coli* ATCC 25922 was used for quality control.

### 4.2. Screening of Aminoglycoside-Resistant Isolates and 16S rRNA Methytransferase Gene Detection

All isolates were screened for high-level resistance to amikacin, gentamicin and apramycin by the Kirby–Bauer disc diffusion method. Isolates that exhibited no zone of inhibition around 15 µg apramycin discs (Oxoid, Basingstoke, UK) or 30 µg amikacin and 10 µg gentamicin discs (BD, Sparks, MD, USA) were further confirmed by gradient strip MIC (bioMérieux, Marcy l’Étoile, France) [2]. When they showed an MIC > 256 mg/L to amikacin and gentamicin, indicating N7-G1405 methyltransferase production, or MIC > 256 mg/L to apramycin, indicating the N1-A1408 enzyme, a multiplex PCR to detect *armA*, *rmtA-F* and *npmA* [37,38,39] was performed as described in Table S5.

### 4.3. Detection of β-Lactamase and Plasmid-Mediated Quinolone Resistance Genes

16S rRNA methyltransferases-positive isolates were further examined for associated β-lactamase and plasmid-mediated quinolone resistance genes. Class A, class B, and class D carbapenemase genes were detected with primer pairs from prior publications [40,41,42,43,44] used in previously established multiplex PCR [45], with the PCR conditions detailed in Supplementary Table S6. *bla*_CTX-M_ genes were detected as described previously [46]. For further differentiation of the allelic variants, the amplicons were purified using the Agencourt AMPure XP beads (Beckman Coulter, Fullerton, CA, USA) and sequenced with the GeXP Genetic Analysis System (Beckman Coulter, Fullerton, CA, USA). *bla*_OXA-1/2/9/10-like_ genes [47] were screened with a protocol listed in Supplementary Table S7. Plasmid-mediated AmpC β-lactamase genes were detected as previously published [48]. PMQR determinants (*qnrA*, *qnrB*, *qnrC*, *qnrD*, *qnrS*, *qepA*) [49,50,51,52] were detected as described in Supplementary Table S8. Identification of the *aac(6′)-Ib-cr* acetyltransferase gene was performed as previously reported [53].

### 4.4. Conjugation Experiments and PCR-Based Replicon Typing

Transferability of 16S rRNA methyltransferase and carbapenemase genes was examined by mating on filters with the sodium azide-resistant *E. coli* J53 recipient strain as previously described [54]. Transconjugants were selected on Luria–Bertani agar containing sodium azide (100 mg/L) supplemented with either amikacin (50 mg/L) and gentamicin (50 mg/L) or 0.5 mg/L meropenem. Transconjugants were confirmed by susceptibility testing and PCR assays for methytransferase and co-transferred resistance genes. In addition, a singleplex PCR for the detection of *aadA2* was performed using previously published primers [55] with a protocol described in Supplementary Table S9.

Incompatibility typing of the plasmids carrying 16S rRNA methyltransferases genes was performed by PCR-based replicon typing (PBRT), involving a combination of multiplex and singleplex PCR panels targeting 21 plasmid replicons as detailed in a previous study [56].

All PCR assays were carried on a Gentier E96 Real-time PCR Sysetem (Xi’an Science and Technology Co., Ltd., Xi’an, China). All amplicons were visualized on a QIAxcel Advanced high-resolution capillary electrophoresis system (Qiagen, Hilden, Germany).

### 4.5. Typing of the 16S rRNA Methytransferase-Producing Isolates

16S rRNA methytransferase-positive isolates of *K. pneumoniae*, *P. mirabilis*, *E. cloacae* complex and S. marcescens, which were selectively sequenced, were subjected to molecular typing to delineate the clonality of the different isolates. *K. pneumoniae* isolates were typed using multi-locus variable number of tandem repeats analysis (MLVA8+) [57]. *P. mirabilis* isolates were typed using the Dienes test [58,59] and the unique strains were further characterized by pulsed field gel electrophoresis (PFGE) using the restriction enzymes ApaI [20]. The *E. cloacae* complex and *S. marcescens* isolates were typed by using the BOX-PCR fingerprinting technique, as detailed in a previous study [60].

### 4.6. Whole-Genome Sequencing

Genomic DNA extraction for PCR and whole-genome sequencing was performed using the PureLink™ Genomic DNA Mini Kit (Thermo Fisher Scientific, Missouri, TX, USA) adhering to the manufacturer’s guidelines, with all homogenization steps carried out by pipetting.

In total, 58% (87/150) of the 16S-RMTase-producing *Enterobacterales* were subjected to whole-genome sequencing. This included all isolates of *E. coli* (*n* = 15), the *C. freundii* complex (*n* = 13), the *K. oxytoca* complex (*n* = 6), *M. morganii* (*n* = 5), *K. aerogenes* (*n* = 3), and *P. stuartii* (*n* = 1). Additionally, selected isolates of *K. pneumoniae* (21/31), *E. cloacae* complex (13/27), *S. marcescens* (9/27), and one representative strain of the *P. mirabilis* clone were included. Short-read sequencing was performed using an Illumina DNA Prep kit (Illumina, San Diego, CA, USA) on a MiSeq V3 (2 × 300 bp) or NextSeq 550 with V2.5 (2 × 150 bp) mid-output flow cell (Illumina, San Diego, CA, USA). Additionally, DNA samples from KP146, KP448, KP3112, KP3161 and KP3648 were sequenced on a MinION Mk1C using the Rapid Barcoding Kit 96 (SQK-RBK110.96) and FLO-MIN106D (R9.4.1) (Oxford Nanopore Technologies, Oxford, UK) with minor adjustments. The final library pool size selection was carried out using 0.4× SPRI magnetic particles to exclude fragments smaller than 1.5 kb [61].

### 4.7. Bioinformatic Analysis

The quality of raw sequencing reads was evaluated using FastQC v0.11.9 (https://www.bioinformatics.babraham.ac.uk/projects/fastqc, accessed on 20 August 2024). Quality trimming and filtering for short and long reads were conducted with fastp v0.23.2 [62] and filtlong v0.2.1 (https://github.com/rrwick/Filtlong, accessed on 20 August 2024). Short-read assemblies were generated with Unicycler v0.4.8 [63]. Long-read assemblies were created with Trycycler v0.5.3 [64] and polished with MEDAKA v1.7.3 (ONT, https://github.com/nanoporetech/medaka, accessed on 20 August 2024). In silico identification was performed with rMLST [65]. Assemblies were annotated with Bakta v 1.7 [66] (database v5.0-full). Antimicrobial resistance determinants and phenotype prediction were carried out using AMRFinderPlus v3.11.4 [67] and ResFinder v4.3.1 [68] (database v2022-05-24). Plasmid analysis was conducted using PlasmidFinder v2.1 with the database v2023-01-18 [69]. MLST profiles were inferred from the assembled genomes using mlst v2.23.0 (Seemann T, mlst Github, https://github.com/tseemann/mlst, accessed on 20 August 2024).

Phylogenetic trees for all individual species, except for *K. pneumoniae*, were constructed using PhaME v1.0.4 [70], considering only SNPs within the coding regions of the core genome. For *K. pneumoniae*, as not all isolates were subjected to WGS, we used multi-locus variable number of tandem repeats analysis (MLVA) for phylogenetic purposes. It was performed according to the MLVA8+ method as previously described [57]. MLVA types were then used for phylogenetic tree construction according to Grissa et al. [18]. All resulting phylogenetic trees were linked to antimicrobial resistance patterns and visualized using iTOL v6.8.1 [71].

## 5. Conclusions

We conducted a retrospective genomic study of 150 consecutive 16S rRNA methyltransferase-producing *Enterobacterales*, collected between 2006 and 2023, to clarify the transmission dynamics of methytransferase and associated antibiotic resistance genes using PCR, WGS and conjugation experiments. We found that the *armA*, *rmtB* and *rmtF* methyltransferase genes were carried by different plasmid Inc types (IncL/M, IncA/C, IncR, IncT, IncFIB, and IncFII), suggesting diverse origins and sources of acquisition due to the large number and variety of host organisms. We showed that persistence of the *armA* gene was associated with the spread of IncL/M-type conjugative plasmids, whose broad-host range included thirteen of the species presented in this study. It was observed that *armA*-carrying IncL/M plasmids also harbor *bla*_CTX-M-3_ or *bla*_CTX-M-15_. Furthermore, carbapenemase (*bla*_NDM-1_, *bla*_VIM-4_, *bla*_VIM-86_, *bla*_OXA-48_), PMQR (*aac(6′)-Ib-cr*, *qnrS*, *qnrB*), and other β-lactamase (*bla*_CMY-4_, *bla*_OXA-1_, *bla*_OXA-10_) genes were also found on these plasmids. Long-read sequencing of ST6260 *K. pneumoniae* isolates revealed a novel resistance association of RmtF1 and RmtB1 methyltransferases, NDM-5 and OXA-232 carbapenemases, and SFO-1 ESBL. The *rmtB1* and *bla*_NDM-5_ were found on the chromosome; *bla*_OXA-232_ was carried by conjugative plasmids of the ColKP3 type, while *rmtF1* was hosted on self-transmissible IncFIB and IncFII plasmids, and was co-transferred with the *bla*_SFO-1_ ESBL gene. The genetic plasticity and adaptability of plasmids containing methyltransferase genes suggest that, by acquiring more potent resistance genes, they have the potential to minimize the continuing threat to public health from 16S rRNA methyltransferase-producing *Enterobacterales*.

## Figures and Tables

**Table 1:** Characteristics of transconjugants harboring 16S rRNA methyltransferase and carbapenemase genes.

Transconjugants from Indicated Donors (No.) *	Transferred Resistance Genes and Plasmid Incompatibility Group		
	16S RMTase Gene	Associated Resistance Genes	Replicon (s)
Transconjugants from donors harboring *armA*, *bla*_CTX-M-3_ and *bla*_NDM-1_			
*S. marcescens* 4487	*armA*	*bla*_CTX-M-3_, *aadA2*	IncL/M
*S. marcescens* 4949	*armA*	*bla*_CTX-M-3_, *bla*_NDM-1_, *aadA2*, *qnrB9*	IncL/M
*C. freundii* (3) *	*armA*	*bla*_CTX-M-3_, *bla*_NDM-1_, *aadA2*, *qnrB9*	IncL/M
*E. coli* 52491	*armA*	*bla*_CTX-M-3_, *bla*_NDM-1_, *bla*_CMY-4_, *aadA2*, *qnrB9*	IncL/M
*E. coli* 52492	*armA*	*bla*_CTX-M-3_, *bla*_OXA-1_, *aac(6′)-Ib-cr*	IncL/M
	*-*	*bla*_NDM-1_, *qnrB9*	IncA/C
*K. pneumoniae* 740	*armA*	*bla*_CTX-M-3_, *bla*_NDM-1_, *aadA2*, *aac(6′)-Ib-cr*, *qnrS1*	IncL/M
Transconjugant from donor harboring *armA*, *bla*_CTX-M-15_ and *bla*_NDM-1_			
*M. morganii* 4395	*armA*	*bla*_NDM-1_, *bla*_OXA-1_, *aadA2*, *qnrB1*	IncT
Transconjugants from donors harboring *armA*, *bla*_CTX-M-3_ and *bla*_VIM-4_			
*S. marcescens* 2942	*armA*	*bla*_CTX-M-3_	IncL/M
*S. marcescens* 1281	*armA*	*bla*_CTX-M-3_	IncL/M
*C. freundii* 2748	*armA*	*bla*_CTX-M-3_, *bla*_VIM-4_	IncL/M
Transconjugant from donor harboring *armA*, *bla*_CMY-4_ and *bla*_VIM-86_			
*P. stuartii* 3347	*armA*	*bla*_VIM-86_, *bla*_CMY-4_, *bla*_OXA-1_, *aac(6′)-Ib-cr*	IncA/C
Transconjugant from donor harboring *armA*, *bla*_CTX-M-15_ and *bla*_oxa-48_			
*E. hormaechei* 3113	*armA*	*bla*_OXA-48_, *bla*_OXA-1_, *aadA2*, *aac(6′)-Ib-cr*, *qnrB1*	IncL/M
Transconjugants from donors harboring *rmtB*, *rmtF*, *bla*_NDM-5_, *bla*_OXA-232_ and *bla*_SFO-1_			
*K. pneumoniae* (2) *	*rmtF*	*bla*_SFO-1_	IncFIB, IncFII
*K. pneumoniae* 3161	*rmtF*	*bla*_SFO-1_, *bla*_OXA-9_	IncFIB
*K. pneumoniae* 3112	*rmtF*	*bla*_OXA-9_	IncFIB
*K. pneumoniae* (4) *	-	*bla*_OXA-232_	Col

* The number of transconjugants obtained, with identical PCR results within each species, is shown in brackets. Further information for each transconjugant can be found in Table S3.

**Table 2:** Characteristics of transconjugants harboring 16S rRNA methyltransferase genes.

Transconjugants from Indicated Donors (No.)*	Transferred Resistance Genes and Plasmid Incompatibility Group		
	16S RMTase Gene	Associated Resistance Genes	Replicon
Transconjugants from donors harboring *armA* and *bla*_CTX-M-3_			
*K. pneumoniae* (14) *	*armA*	*bla*_CTX-M-3_, *aadA2*	IncL/M
*K. pneumoniae* (3) *	*armA*	*bla*_CTX-M-3_, *bla*_OXA-10_, *aac(6′)-Ib-cr*	untypable
*K. pneumoniae* 1698	*armA*	*bla*_CTX-M-3_	IncL/M
*E. hormaechei* (14) *	*armA*	*bla*_CTX-M-3_, *aadA2*	IncL/M
*E. hormaechei* (7) *	*armA*	*bla*_CTX-M-3_, *bla*_OXA-1_, *aac(6′)-Ib-cr*	IncL/M
*E. hormaechei* (2) *	*armA*	*bla*_CTX-M-3_	IncL/M
*E. hormaechei* 401	*armA*	*bla*_CTX-M-3_, *aadA2*, *bla*_OXA-1_, *aac(6′)-Ib-cr*, *qnrB1*	IncL/M
*E. asburiae* 146	*armA*	*bla*_CTX-M-3_, *aadA2*	IncL/M
*S. marcescens* (14) *	*armA*	*bla*_CTX-M-3_, *aadA2*	IncL/M
*S. marcescens* 2921	*armA*	*bla*_CTX-M-3_	IncL/M
*S. marcescens* 5327	*armA*	*bla*_CTX-M-3_	IncA/C
*S. marcescens* 3247	*armA*	*bla*_CTX-M-3_, *bla*_OXA-1_, *aac(6′)-Ib-cr*	IncL/M
*E. coli* (8) *	*armA*	*bla*_CTX-M-3_, *aadA2*	IncL/M
*C. freundii* (2) *	*armA*	*bla*_CTX-M-3_, *aadA2*	IncL/M
*C. freundii* (2) *	*armA*	*bla*_CTX-M-3_, *bla*_OXA-1_, *aac(6′)-Ib-cr*	IncL/M
*C. freundii* 1739	*armA*	*bla*_CTX-M-3_, *aadA2*, *qnrB38*	IncL/M
*C. freundii* 3757	*armA*	*bla*_CTX-M-3_, *bla*_OXA-1_, *aac(6′)-Ib-cr*, *qnrB9*	IncL/M
*C. portucalensis* 2648	*armA*	*bla*_CTX-M-3_, *aadA2*	IncL/M
*K. michiganensis* (3) *	*armA*	*bla*_CTX-M-3_, *aadA2*	IncL/M
*K. oxytoca* 1673	*armA*	*bla*_CTX-M-3_, *aadA2*	IncL/M
*K. oxytoca* 1765	*armA*	*bla*_CTX-M-3_, *bla*_OXA-1_, *aac(6′)-Ib-cr*	IncL/M
*M. morganii* 1990	*armA*	*bla*_CTX-M-3_, *aadA2*	IncL/M
*M. morganii* 2789	*armA*	*bla*_CTX-M-3_	IncL/M
*M. morganii* 2117	*armA*	*bla*_CTX-M-3_, *bla*_OXA-1_, *aac(6′)-Ib-cr*	IncL/M
*K. aerogenes* (3) *	*armA*	*bla*_CTX-M-3_, *aadA2*	IncL/M
Transconjugants from donors harboring *armA* and *bla*_CTX-M-15_			
*K. pneumoniae* 2010	*armA*	*bla*_CTX-M-15_, *aadA2*	IncL/M
*K. pneumoniae* 510	*armA*	*bla*_CTX-M-15_, *aadA2*	untypable
*K. pneumoniae* 727	*armA*	*bla*_CTX-M-15_, *bla*_OXA-1_, *aac(6′)-Ib-cr*	untypable
*K. pneumoniae* 2280	*armA*	*bla*_CTX-M-15_, *bla*_OXA-10_, *aac(6′)-Ib-cr*, *qnrB1*	untypable
*K. pneumoniae* 2158	*armA*	*bla*_CTX-M-15_, *bla*_OXA-1_, *aadA2*, *aac(6′)-Ib-cr*	IncR
*S. marcescens* 3560	*armA*	*bla*_CTX-M-15_, *aadA2*	IncL/M
*C. freundii* 979	*armA*	*bla*_CTX-M-15_	IncL/M
*C. freundii* 2739	*armA*	*bla*_CTX-M-15_, *aac(6′)-Ib-cr*	IncFIB
*K. michiganensis* 2117	*armA*	*bla*_CTX-M-15_, *bla*_OXA-1_, *aac(6′)-Ib-cr*	IncL/M

* The number of transconjugants obtained, with identical PCR results within each species, is shown in brackets. Further information for each transconjugant can be found in Table S3.

## Data Availability

All data generated during this study are included in this article and its Supplementary Materials. Relevant links and references to additional sources are provided in the text. Draft genome sequences have been uploaded to the National Center for Biotechnology Information (NCBI) database and are accessible under BioProject IDs PRJNA1077246 and PRJNA1132711. The complete genome sequences of the ST6260 *K. pneumoniae* isolates (KP146, KP448, KP3112, KP3161, and KP3648) are available under BioProject ID PRJEB70839.

## Supplementary Materials

The following supporting information can be downloaded at https://www.mdpi.com/article/10.3390/antibiotics13100950/s1: Table S1: Properties of 16S rRNA methytransferase-producing *Enterobacterales* isolates collected from 2006 to 2023; Table S2: Prevalence of 16S rRNA methyltransferase producers among different *Enterobacterales* species; Table S3: Characteristics of transconjugants harboring 16S rRNA methyltransferase genes; Table S4: Antimicrobial susceptibility of 16S rRNA methytransferase-producing *Enterobacterales* isolates; Table S5: 16S rRNA methyltransferase (16S-RMTase) mPCR; Table S6: Carbapenemase mPCR; Table S7: OXA mPCR; Table S8: Plasmid-mediated quinolone resistance (PMQR) mPCR; Table S9: *aadA2* PCR.
